# Paradoxical puborectalis syndrome on diffusion-weighted imaging: a retrospective study of 72 cases

**DOI:** 10.1038/s41598-017-03127-8

**Published:** 2017-06-07

**Authors:** Guiqin Liu, Zhe Cui, Yongming Dai, Qiuying Yao, Jianrong Xu, Guangyu Wu

**Affiliations:** 1 Department of Radiology, Renji Hospital, School of Medicine, Shanghai Jiao Tong University, Shanghai, China; 2 Department of Gastrointestinal Surgery, Renji Hospital, School of Medicine, Shanghai Jiao Tong University, Shanghai, China; 3 Magnetic Resonance Imaging Institute for Biomedical Research, Wayne State University, Detroit, MI USA

## Abstract

This study aimed to evaluate the application value of diffusion-weighted imaging (DWI) for assessing paradoxical puborectalis syndrome (PPS) in patients with obstructive defecation syndrome (ODS). The medical records of 72 ODS patients who underwent magnetic resonance (MR)-DWI and MR-defecography were retrospectively reviewed. The differences in the apparent diffusion coefficients (ADCs) and the thickness of the right and left branches of the puborectalis muscles between the PPS(+) and PPS(−) groups were compared. In addition, the absolute within-patient differences between the right and left branches (ADC, thickness) were compared between the two groups. The absolute difference in ADCs (right branch - left branch) was significantly different between the two groups. Regardless of whether the ADC was acquired through single-ROI (0.10 ± 0.08 vs 0.23 ± 0.18, P = 0.000) or multi-ROI (0.16 ± 0.14 vs 0.27 ± 0.17, P = 0.009) analysis, the PPS(+) patients displayed a lower absolute ADC difference than did the PPS(−) patients. However, there was no statistically significant difference in the ADC value, thickness or the absolute difference in thickness between the two groups. These findings suggest that DWI may have value in quantitatively assessing the puborectalis muscle in ODS patients, whereas the value of puborectalis thickness in such aspect needs further study.

## Introduction

Paradoxical puborectalis syndrome (PPS) comprises clinical signs and symptoms related to a disorder of the puborectalis muscle that prevents the normal evacuation of faeces and prevents the anorectal angle from opening during defecation. This results in obstructive defecation syndrome (ODS), which includes difficulty in faeces expulsion, straining during defecation for more than 25% of the time, prolonged time spent on the toilet, hard faeces and the need for self-digitation; consequently, ODS substantially diminishes the quality of life of affected patients^1^.

To date, imaging modalities have rarely been used to assess PPS, and most of their applications have focused on the morphological features of the disease^1, 2^. However, evaluating the morphological features of PPS is not sufficient because the results of this type of examination can be influenced by many factors. For example, magnetic resonance (MR) defecography requires a high degree of cooperation from the patient^1^, and it is difficult to assess disease severity using morphological features. Imaging modalities that enable the quantitative evaluation of the disease may have the potential to improve this situation. Because PPS mainly involves the puborectalis muscle, functional imaging of the disease may be feasible, and MR diffusion-weighted imaging (MR-DWI) can provide useful information that can be used to quantitatively reflect tissue microstructure and pathophysiology. The purpose of this study was to evaluate the value of imaging characteristics, including the thickness and MR-DWI parameters, of the puborectalis in assessing PPS in patients with ODS. To the best of our knowledge, no previously published studies have explored this topic.

## Results

### Patient characteristics

The characteristics of the PPS patients and normal patients are shown in Table 1. A total of 72 patients who met the study criteria, including 26 men and 46 women with a mean age of 54.1 ± 14.5 years (range, 14–77 years), were included in the study. Subjects were divided into a PPS(+) group (n = 29) and a PPS(−) group (n = 43). One woman had undergone a total hysterectomy without oophorectomy for early stage uterine cervical cancer. There were no differences in age, gender or pelvic-related surgery between the PPS(+) and PPS(−) groups (Table 1). All of the subjects were successfully evaluated, and satisfactory images were obtained.

**Table 1:** Characteristics of paradoxical puborectalis syndrome patients and control patients.

			PRS(+)	PRS(−)	*P* value
No			29	43	/
Age, years (mean ± SD)			54.72 ± 11.96	53.74 ± 16.14	0.768
Gender (M/F)			10/19	16/27	0.813
pelvic-related surgery (+/−)			0/29	1/42	1.000
ADC (×10^−3^ mm^2^/s)	R	s-ROI	1.26 ± 0.21	1.27 ± 0.24	0.738
		m-ROIs	1.32 ± 0.25	1.32 ± 0.23	0.946
	M	s-ROI	1.25 ± 0.29	1.29 ± 0.30	0.542
		m-ROIs	1.30 ± 0.32	1.41 ± 0.29	0.095
	L	s-ROI	1.21 ± 0.19	1.16 ± 0.28	0.281
		m-ROIs	1.21 ± 0.14	1.25 ± 0.27	0.424
Puborectalis thickness (mm)	R		6.50 ± 1.38	5.99 ± 1.81	0.202
	M		7.61 ± 2.45	7.37 ± 2.30	0.671
	L		6.41 ± 1.36	6.16 ± 1.93	0.552
ADC(|R−L|) (×10^−3^ mm^2^/s)*	s-ROI		0.10 ± 0.08	0.23 ± 0.18	0.000
	m-ROIs		0.16 ± 0.14	0.27 ± 0.17	0.009
AUC to differentiate PBS + from PBS−	s-ROI		0.735		0.001
	m-ROIs		0.714		0.002
Puborectalis thickness(|R−L|) (mm)*			0.39 ± 0.39	0.45 ± 0.38	0.556

ADC: apparent diffusion coefficient; s-ROI: single ROI; m-ROI: multi ROIs; ADC(|R−L|): the absolute difference in ADCs between the right and left branches; puborectalis thickness(|R−L|): the absolute difference in puborectalis thickness between the right and left branches; AUC: area under the curve. *Mann-Whitney test.

### Inter- and intra-observer reproducibility

**Figure 1:**
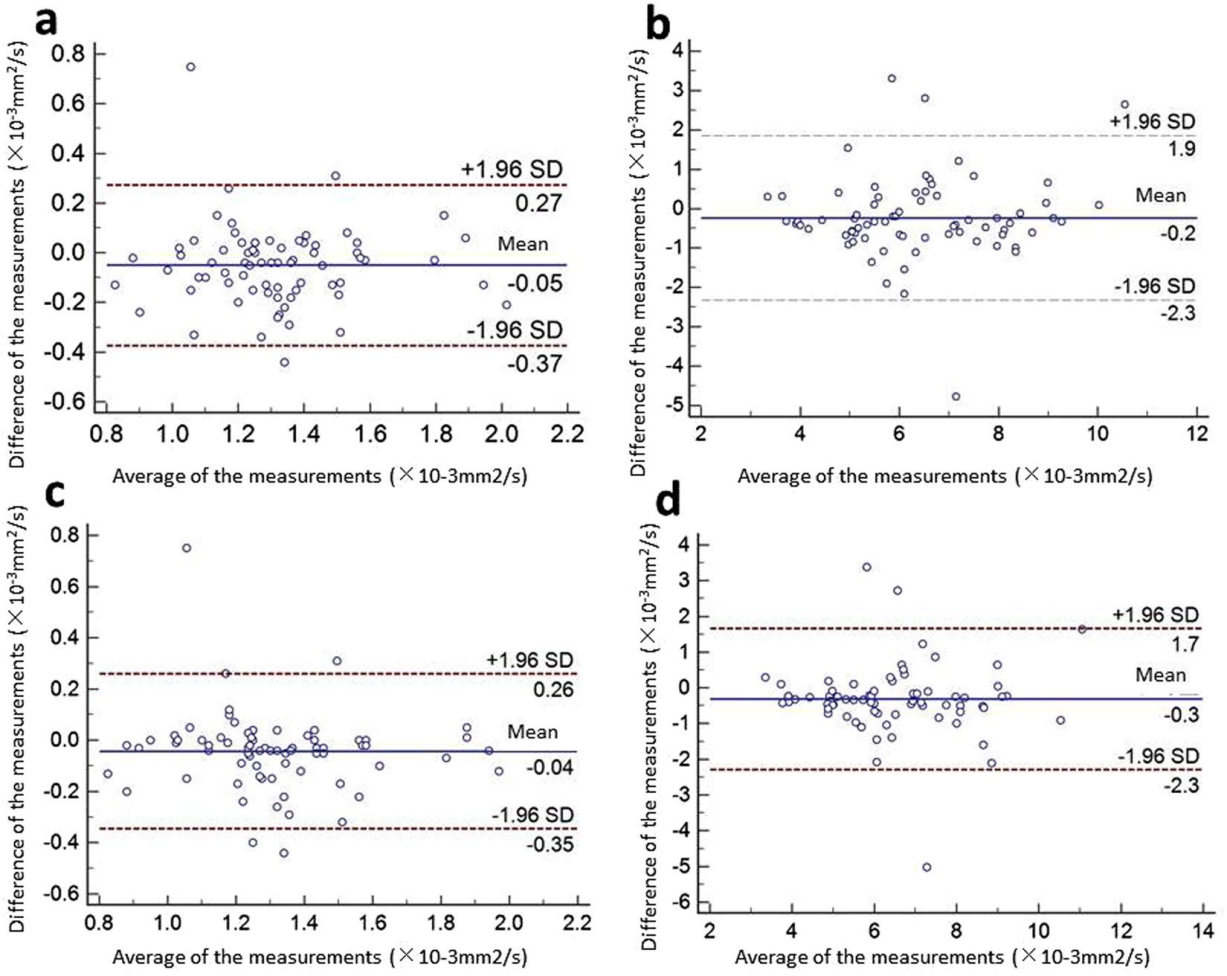
Bland-Altman plots of inter- and intra-observer agreement. The calculation of ADCs (**a**) and puborectalis thickness (**b**) between radiologists A and B; the calculation of ADCs (**c**) and puborectalis thickness (**d**) for the same radiologist. The solid lines indicate the mean of the differences between measurements. The dashed lines indicate the 95% limits of agreement, defined as the mean difference ±1.96 (standard deviation) of the differences.

The intra-class correlation coefficients (ICCs) and their 95% confidence intervals (CIs) between the two radiologists were 0.87 (0.80, 0.92) and 0.89 (0.82, 0.93) for apparent diffusion coefficients (ADCs) and puborectalis thickness, respectively, whereas those for the same radiologist were 0.90 (0.83, 0.93) and 0.91 (0.85, 0.94) for ADCs and puborectalis thickness, respectively. All ICCs were greater than or equal to 0.82, demonstrating almost perfect agreement both between and within radiologists. We generated Bland-Altman plots to facilitate comparisons between the two radiologists or within each radiologist to calculate ADCs and puborectalis thickness (Fig. 1). Points in the Bland-Altman plots tended to distribute symmetrically and uniformly around the line representing the mean difference.

### Inter-method agreement

The ICCs and their 95% CIs between the two applied methods for calculating ADCs of the right branch, midpoint and left branch were 0.83 (0.72, 0.89), 0.89 (0.83, 0.93), and 0.83 (0.72, 0.89), respectively. All of the ICCs were greater than or equal to 0.83, demonstrating excellent agreement between the two methods.

### Imaging results

The statistical analyses showed no significant differences in ADCs at each point between the PPS(+) and PPS(−) groups; however, the absolute difference in ADCs between the right and left branches differed significantly between the two groups. Regardless of whether the ADC value was acquired through a single-ROI (0.10 ± 0.08 vs 0.23 ± 0.18, P = 0.000) or multi-ROI (0.16 ± 0.14 vs 0.27 ± 0.17, P = 0.009) analysis, the PPS(+) patients displayed a lower absolute ADC difference than did the PPS(−) patients, and the area under the curve (AUC) was (0.735, P = 0.001), (0.714, P = 0.002) in the ROC analysis. The threshold values of ADCs (|R-L|) to differentiate PBS(+) from PBS(−) were 0.235 × 10^–3^ mm^2^/s (sensitivity: 46.5%; specificity: 93.1%) and 0.255 × 10^–3^ mm^2^/s (sensitivity: 46.5%; specificity: 89.7%) for the single- and multi-ROI analyses, respectively. The mean thickness of the puborectalis in the PPS(+) group was generally greater than in the PPS(−) group, but the difference between groups was not statistically significant. The absolute difference in thickness (between the two branches) was not significantly different between the two groups (0.39 ± 0.39 vs 0.45 ± 0.38, P = 0.556). In addition, in the patients in the PPS(−) group, the right side of the puborectalis was thinner than the left side, with mean thicknesses of 5.99 ± 1.81 mm and 6.16 ± 1.93 mm, respectively.

## Discussion

Among the factors leading to outlet obstruction in adults, pelvic floor dysfunction accounts for more than 50% of the cases^3^, and PPS is a critical factor. With the growing body of research, understanding of this disease has gradually improved. In particular, it has become clear that in PPS patients, infections and other complications stimulate hypertrophy of the muscle fibres, resulting in a loss in the ability of the puborectalis muscle to relax during defecation and, paradoxically, to retract.

Imaging modalities are of great importance in evaluating PPS. An ideal evaluation method would allow clinicians to accurately diagnose the disease and determine its severity, which would help in determining an optimized individual patient treatment plan and avoiding unnecessary therapy, expense, and treatment delays. However, imaging evaluations based on morphological features are not sufficient, and the absence of a quantitative evaluation method is a challenge. MR-DWI is a non-invasive functional imaging method that can be used to image the Brownian motion of water molecules. Water molecular diffusion in tissues is not free and reflects the interaction of water molecules with many obstacles; therefore, diffusion patterns can reveal microscopic details about tissue architecture. By calculating the ADCs, quantitative information on diffusion coefficients can be obtained. This technique is increasingly being used to assess diseases and monitor treatment effects^4^. However, to our knowledge, the value of DWI in assessing PPS has not been determined.

Our results showed no significant difference in ADCs between the PPS(+) and PPS(−) groups when the left and right branches of the puborectalis were evaluated independently. However, when the absolute difference in ADCs between the left and right branches of the puborectalis were compared, regardless of whether ADCs were acquired through single-ROI or multi-ROI analyses, we found significant difference between the two groups. Patients in the PPS(+) group displayed a lower absolute difference in ADCs than did patients in the PPS(−) group. Within subjects, the ADC of the right branch was higher than that of the left branch, and the absolute difference in ADCs in the PPS(+) group may have been the result of either decreased ADCs of the right branch or increased values of the left branch. Considering that muscle fibre hypertrophy is an important abnormal pathology of the disease and that it consistently leads to a decrease in the ADC^5^, we speculate that a decrease in the ADC of the right branch may be the main reason for our results. Our results also indicate the puborectalis abnormality may be unilateral. If this assumption can be confirmed via pathological analysis, it will be a critical piece of information for selecting the appropriate treatment plan. In addition, considering that the ADC of a single branch did not differ between the patient groups, we speculate that the base value of the ADC in the puborectalis may vary among patients and that by analysing the absolute difference in ADC, we were able to control for such variation in the ADC among patients, which might also explain our findings.

PPS often includes puborectalis spasms or hypertrophy; therefore, puborectalis thickness might be used to evaluate its development because changes in thickness may affect the strength of muscle contractions. Although the mean thickness of the puborectalis in the PPS(+) group was generally greater than that in the PPS(−) group, neither the thickness nor the absolute difference in thickness differed significantly between the two groups. Based on our research and clinical experience, some patients display no change in the thickness of the muscle during imaging, but nonetheless have severe clinical symptoms. Therefore, we hypothesized that measuring the thickness of the puborectalis is inadequate for assessing abnormalities in PPS as pathophysiological changes or microstructural damage of the muscle might occur prior to alterations in size. Previously, published articles^6, 7^ have shown that the right side of the puborectalis is consistently thinner than the left side. In our study, such results were not obvious. This may be because during the course of the disease, changes in muscle morphology or thickness are not identical between the two sides.

Currently, dynamic defecation proctography and dynamic MRI play key roles in the assessment of PPS. Dynamic defecation proctography is the current gold standard for posterior compartment imaging^8^, but this method cannot display the anatomical structures of the puborectalis itself or of the adjacent tissue. Dynamic MRI solves this problem and improves the ability to evaluate the posterior compartment^9, 10^. However, the strict requirements for patient cooperation and the lack of quantitative information are shortcomings of this method. In our study, we demonstrated a method to quantitatively evaluate the puborectalis with high intra- and inter-observer agreement. This method meets the basic requirements for practical use and may have the potential to reflect changes in organizational structure and the microenvironment. It might also be a useful imaging modality to supplement conventional methods and improve the evaluation of PPS.

There were several limitations to this study. First, although this study provides evidence of a difference between PPS(+) and PPS(−) patients in the absolute difference in ADC between the right and left puborectal branches, our results are not supported by strictly pathological results because it is not feasible to obtain results of pathological changes for most patients during clinical procedures. Second, because this was a preliminary study, the number of patients in the PPS(+) group was small and was insufficient for evaluating the true value of DWI for improving the diagnosis and severity assessment of PPS based on quantitative data. Furthermore, additional detailed analyses, such as of the correlations between ADC and clinical signs/indexes were not performed. In addition, although we consider an abnormal puborectalis muscle to primarily involve the the right branch, this assumption is based on theoretical knowledge, and we have no evidence to confirm whether variations in ADCs will affect the results. Due to these limitations, the true value of DWI for evaluating PPS needs to be determined by conducting additional studies with larger populations and more complete pathological evidence. We aim to perform such studies in the future. In the future, PPS may be treated in a more sophisticated and efficacious manner than it is presently. Therapies have not been optimized, and many different surgical procedures and nonsurgical therapies have been reported for the treatment of this condition. DWI may provide valuable information on the puborectalis muscle, including information on congenital variations, that might assist clinicians in selecting the optimal treatment, evaluating the prognosis and following-up the disease.

## Conclusion

Our findings suggest that DWI may have value in quantitatively assessing the puborectalis muscle in ODS patients; however, the utility of puborectalis thickness in such an assessment requires further study. It may be due to pathophysiological changes or microstructural damage of the muscle that occur prior to alterations in size.

## Materials and Methods

### Study population

This study was approved by the Medical Ethical Committee of the Renji Hospital, School of Medicine, Shanghai Jiao Tong University. Written informed consent was obtained from each subject who participated in the study. The methods were carried out in accordance with the approved guidelines.

The medical records and imaging series of 72 consecutive patients treated for symptoms of ODS between February 2015 and February 2017 were evaluated for this study. ODS symptoms were diagnosed by the referring physician in all cases on the basis of medical history and clinical examinations. We used the Rome III criteria^11^ to exclude patients with symptoms suggestive of constipation not secondary to ODS (i.e., lumpy stools, stools rarely loose without laxatives and fewer than three defecations per week). We also excluded patients with irritable bowel syndrome (IBS), which was defined as recurrent abdominal pain and/or discomfort 3 days/month for the past 3 months associated with an improvement in defecation and a change in stool frequency and/or stool form at onset; exclusion criteria included patients with the IBS-C subtype. Additional investigations included an anorectal manometry, ano-proctoscopy and electromyography or biopsy as needed.

### Imaging Protocol

Magnetic resonance imaging (MRI) was performed according to a standard protocol using a 3.0 T permanent field (Ingenia, Philips, Netherlands). All examinations were performed by the same radiologist, who was trained in the dynamic imaging of pelvic floor dysfunctions using dynamic pelvic MRI. Immediately prior to the study, the patients were carefully coached by two skilled technicians. A standard interactive imaging technique was used as follows. While lying on the left side, the patient was positioned on the MRI gantry with the knees flexed, and a proof pad was placed beneath the exposed buttocks to collect any material. A rectal tube was inserted through the anus, and 150 ml of ultrasound gel was injected through the tube as the rectal contrast medium.

The MRI protocol began with a T1-weighted localizer sequence with a large field of view to identify the midline sagittal section. This was followed by T2-weighted static images of the pelvic region acquired in the axial, coronal and sagittal planes to provide a complete anatomic evaluation using a driven equilibrium fast spin echo pulse sequence (TR/TE, 2000/80 ms; FA, 90°; FOV, 35 cm; slice thickness, 4 mm; interslice gap, 1 mm; matrix, 292 × 210; NEX, 1; acquisition time, 4.40 min; number (no) of images, 32). DW images were then acquired in the axial plane with a single shot echo-planar sequence (FOV, 320 × 210 mm^2^; matrix, 94 × 80; slice thickness, 3 mm; slices, 80; DW directions, 32; TR/TE, 12736/81 ms; b = 0 mm^2^/s, 700 mm^2^/s; spectral adiabatic inversion recovery for fat suppression; imaging time, 4 min 32 s).

Dynamic images were then obtained at rest, on squeezing and at maximal straining in the midsagittal plane using a T1-weighted radio frequency spoiled steady-state acquisition rewound gradient echo pulse sequence (TR/TE, 10/3.7 ms; FA, 60°; FOV, 34 cm; section thickness, 20 mm; interslice gap, 10 mm; matrix size, 256 × 180; BW, 60.0 kHz; NEX, 1; acquisition time, 1.23 min; no of images, 24) and a balanced steady-state acquisition rewound gradient echo pulse sequence (TR/TE, 8.8/4.4 ms; FA, 60°; FOV, 34 cm; section thickness, 20 mm; gap, 10 mm; matrix size, 256 × 180; NEX, 1; acquisition time, 36 s; no of images, 12). The patient was instructed to initiate movement at will and to indicate this by intercom to allow for continuous image acquisition. Using real-time image reconstruction, the examiner could constantly monitor, instructor encourage the patient and ensure the performance of desired manoeuvres.

### Image Analysis

**Figure 2:**
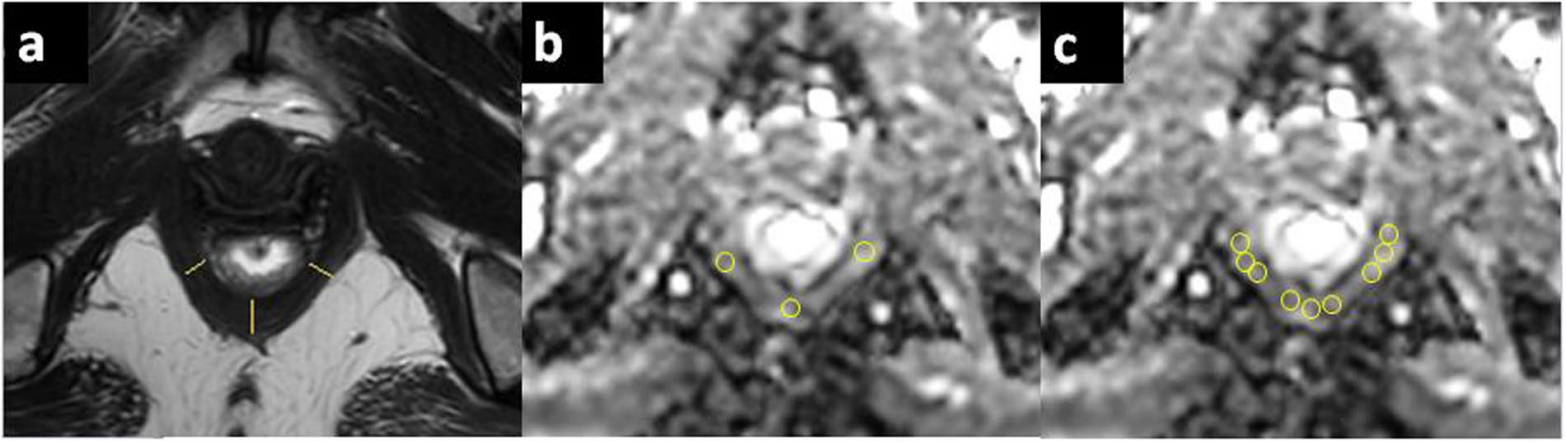
Measurement of puborectalis thickness and ADC. (**a**) Measurement of puborectal thickness where the puborectalis is at its widest (adjacent to the rectal wall at the point where the fibres attach to the pubis; the yellow lines indicate where the puborectal thickness measurements were obtained, right in the middle and at a third of the total length of each branch. (**b**) ROIs were placed at the slice where the puborectalis was widest. An ROI was first placed at the point of the thickest slice of the middle zone (M zone). Then, ROIs were placed at a third of the total length of the left and right branches, close to the M zone. (**c**) Another method for placing ROIs to increase the numbers of voxel is to add two ROIs on both sides of original ROI and average the values of the three ROI to get the final result.

Puborectalis thickness was measured in the axial plane on T2-weighted images at the widest slice adjacent to the rectal wall according to the methods of How *et al*.^12^ with modifications. In total, three points were measured: at the midpoint and at one third of the total length of each branch, (Fig. 2). Considering that PPS includes puborectalis spasms or hypertrophy, we suspected that the abnormality would probably be at the thickest slice. Therefore, our first ROI was at the point of the thickest slice at the midpoint (called the M zone). We then placed ROIs at one third of the total length of the left and right branches, near the M zone, the size of single ROI was (17.7 mm^2^, 7–24 mm^2^). In addition, to increase the number of voxels within the ROIs, we applied a second method the obtain ADCs: we added an ROI along each side of the original ROI and averaged the values of these three ROIs to obtain the final results, the size of the combined ROIs was (53.1 mm^2^, 21–72 mm^2^). The absolute differences between the right and left branches $(|AD{C}_{{\rm{R}}}-AD{C}_{L}|,\,|T{{\rm{h}}{\rm{i}}{\rm{c}}{\rm{k}}{\rm{n}}{\rm{e}}{\rm{s}}{\rm{s}}}_{R}-T{{\rm{h}}{\rm{i}}{\rm{c}}{\rm{k}}{\rm{n}}{\rm{e}}{\rm{s}}{\rm{s}}}_{{\rm{L}}}|)$ were also calculated. All of the data sets were independently analysed, and measurements were performed by two radiologists with 4 to 7 years of experience in pelvic imaging. To prevent bias caused by learning effects, the images were presented in a random order.

### Reference Standard

A previous study^1^ defined PPS as a persistent impression at the posterior aspect of the anorectal junction and a thickening of the muscle during defecation of the rectal contrast medium. External anal sphincter dyssynergia is occasionally observed on the antero-posterior view as a lack of anal canal widening, and a weak barium stream reveals a narrow and largely symmetrical internal lumen. Additional features include prolonged evacuation time, contrast retention after repeated attempts, and associated anterior rectocele sliding below the puborectalis sling.

### Statistical Analysis

Statistical analyses were performed using SPSS 19.0 (SPSS Inc., Chicago, IL, USA). The absolute differences in ADCs and thickness between the left and right branches of the puborectalis were calculated for each patient. Continuous variables are expressed as the mean ± standard deviation. Quantitative variables were compared using independent samples t-tests or Mann-Whitney tests, and categorical data were compared using χ² tests or Fisher’s exact tests as appropriate. A receiver-operator characteristic (ROC) curve analysis was performed^13^, and discriminative power was assessed using the area under the ROC curve (AUC)^14^. A P value of less than 0.05 was considered statistically significant. Inter- and intra-observer agreements and inter-method agreement were assessed using ICCs^15^, and Bland-Altman plots^16^ were constructed for inter- and intra-observer agreement. The 95% CIs associated with the ICCs were calculated. ICC values of 0–0.20, 0.21–0.40, 0.41–0.60, 0.61–0.80, and 0.81–1.00 were considered to indicate poor, fair, moderate, good, and very good agreement, respectively.
